# The Power of Visual Texture in Aesthetic Perception: An Exploration of the Predictability of Perceived Aesthetic Emotions

**DOI:** 10.1155/2018/1812980

**Published:** 2018-09-09

**Authors:** Jianli Liu, Edwin Lughofer, Xianyi Zeng, Zhengxin Li

**Affiliations:** ^1^College of Textiles and Clothing, Jiangnan University, Wuxi 214122, China; ^2^Department of Knowledge-Based Mathematical Systems, Johannes Kepler University Linz, A-4040 Linz, Austria; ^3^Université Lille Nord de France, F-59000 Lille, France; ^4^GEMTEX, ENSAIT, F-59056 Roubaix, France; ^5^School of Information Science and Technology, ShanghaiTech University, Shanghai 201210, China

## Abstract

How to interpret the relationship between the low-level features, such as some statistical characteristics of color and texture, and the high-level aesthetic properties, such as warm or cold, soft or hard, has been a hot research topic of neuroaesthetics. Contrary to the black-box method widely used in the fields of machine learning and pattern recognition, we build a white-box model with the hierarchical feed-forward structure inspired by neurobiological mechanisms underlying the aesthetic perception of visual art. In the experiment, the aesthetic judgments for 8 pairs of aesthetic antonyms are carried out for a set of 151 visual textures. For each visual texture, 106 low-level features are extracted. Then, ten more useful and effective features are selected through neighborhood component analysis to reduce information redundancy and control the complexity of the model. Finally, model building of the beauty appreciation of visual textures using multiple linear or nonlinear regression methods is detailed. Compared with our previous work, a more robust feature selection algorithm, neighborhood component analysis, is used to reduce information redundancy and control computation complexity of the model. Some nonlinear models are also adopted and achieved higher prediction accuracy when compared with the previous linear models. Additionally, the selection strategy of aesthetic antonyms and the selection standards of the core set of them are also explained. This research also suggests that the aesthetic perception and appreciation of visual textures can be predictable based on the computed low-level features.

## 1. Introduction

We all know that stepping carelessly onto a wet marble floor might cause us to slip. The same way, we know that running on grass in the park is safe. Even if we fall we are unlikely to get hurt. Although we may not be aware of it, visual texture provides us with information that triggers certain emotional qualities and expectations. Textures have substantial impact on product design. Designers of architecture, packages, and interfaces of application software will profit from the ability to use texture in a predictable way to evoke some intended emotions and achieve the predefined targets. To investigate how visual texture activates certain emotions and feelings, several models of visual aesthetic perception have been proposed in recent years from the aspects of cognitive neuroscience, and neurobiological and mathematical sciences [1].

The general methods always used in machine learning, such as support vector machine, neural network, and multi-category classifiers, have been employed to recognize and classify images labeled with predefined aesthetic properties [2–7]. Some regression models and genetic algorithm approaches have also been used to predict the aesthetic properties of images by taking low-level statistical textural features as inputs [8]. Although the machine learning models are black boxes with little interpretability, they are general methods for model building and can be easily carried out.

To improve the interpretability, several neuropsychological and neuroaesthetic models of aesthetic perception of visual textures have been proposed [9]. Chatterjee et al. proposed a neuropsychological model for aesthetic appreciation of visual texture by taking the neural underpinning of visual information processing as references [10, 11]. Leder et al. proposed an influential framework of aesthetic experiences and aesthetic judgment in which the flow of information was processed in different circuits like the neural networks of the brain [12, 13]. Koelsch et al. proposed an integrative and neurofunctional model for human emotions, in which four core neural circuits for emotion processing were involved [14]. Redies proposed a unifying model of visual aesthetic experience by combining universal beauty and cultural context [15]. Thumfart et al. proposed a hierarchical feed-forward model to explore the relationship between computational texture features and the aesthetic properties of visual textures using the results of a psychological experiment [16]. And some further research studies have been continued by Liu et al. [17, 18].

This article aims at the development of a method to calculate the degree to which certain aesthetic feelings are associated with a particular texture and an investigative method for the modeling of aesthetic perception of visual textures. After the discussion of related work in Section 1, we propose four methods to extract low-level features to objectively describe the visual texture in Section 2. The semantic differential experiment to collect the predefined aesthetic properties is discussed in Section 3. After the introduction of the proposed aesthetic perception model of visual textures, the modeling methods of beauty appreciation of visual textures using multiple linear or nonlinear regression are detailed in Section 4. The results are discussed in Section 5.

## 2. Low-Level Texture Feature Extraction

### 2.1. Database of Visual Textures

**Figure 1:**
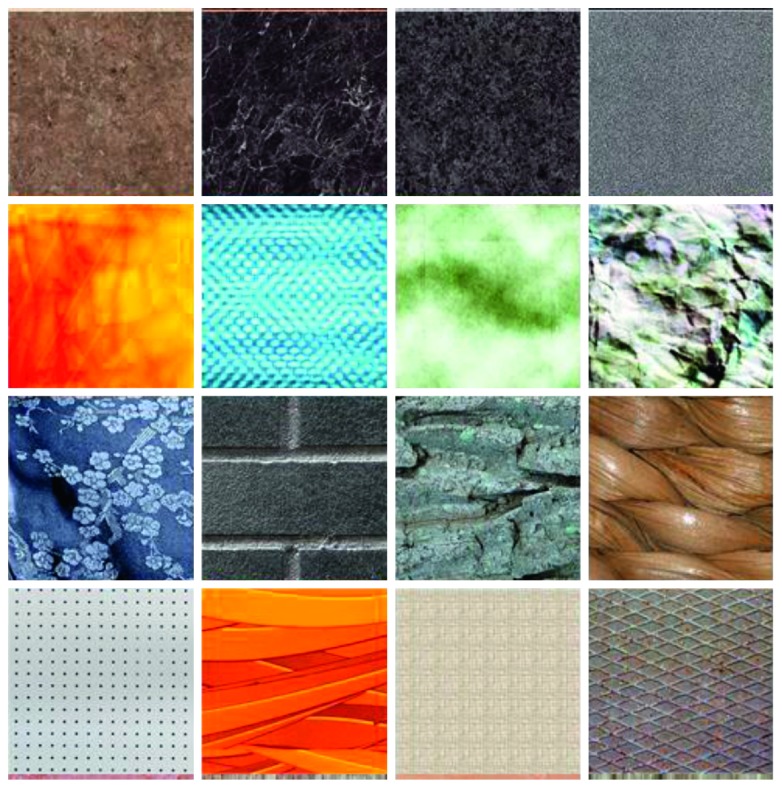
Example textures.

We selected the SynTex database (available on request; see Figure 1 for examples) for experiment materials, which consists of 151 high-quality textural images and has been used in the sixth framework program (NEST 043157) supported by the European Union [16].

### 2.2. Feature Extraction Algorithms

In this section, we just briefly discuss four algorithms to calculate low-level textual features to describe the statistical properties of visual texture. A more elaborate description of these four methods has been provided in a previous study [17–19]. The average of the hue saturation value color matrix elements was calculated after the texture images were transformed into HSV from the RGB space. Thus, three color features were calculated for each visual texture. We modified the two parameters of the gray-level co-occurrence matrix (GLCM), the orientation *θ* or distance *d* between pixels at four different levels, and obtain 64 statistical features for each visual textures. Tamura features [20], coarseness, contrast, and directionality were calculated as characteristics representing the psychological responses to visual perception. More specifically, we calculated *L*^1^ and *L*^2^ norms and Shannon entropy from the high-frequency subbands of the first four levels after wavelet transform proposed by Do and Vetterli [21]. Then, we extracted 36 wavelet signatures from each texture image. In total, 106 features were calculated for each visual texture.

### 2.3. Feature Selection

As mentioned in Section 2.2, each visual texture will be represented by 106 low-level features after texture analysis. However, not all of these features are equally important for model building. Some of them are redundant or even irrelevant. To ensure better performance and control the complexity of the built model, feature selection is one of the effective methods to discover the most valuable and optimal features at very low computational cost [22]. In this paper, we used neighborhood component analysis for robust feature selection prior to model building. Neighborhood component analysis is a well-known, conceptually simple distance metric learning method developed under a well-formulated probabilistic framework with graph label consistency constraints [23]. The top 10 features with larger weights used for model building are listed in Table 1.

## 3. Semantic Differential Experiment

### 3.1. Selection of the Aesthetic Antonyms

To select suitable adjectives to represent the aesthetic meanings conveyed by the visual textures, twenty (10 males and 10 females, age range 16–24 years) undergraduate students of Jiangnan University were recruited. The present study was performed with the approval of the ethical committee of Jiangnan University for experiments with human participants. Before experiment, we introduced the aim of aesthetic antonym selection to all participants with a prepared example. In the example, some pairs of aesthetic antonyms, such as “natural-artificial, random-regular, modern-ancient, hard-soft, and simple-complex,” were listed when a textural image was displayed on a Sharp big PAD. Then, the participants were asked to collect 20 pairs of aesthetic antonyms that are similar to the given examples and can be used to represent human aesthetic feelings when appreciating a textural image. Within 7 days, twenty aesthetic antonyms were collected, as listed in Table 2.

To select the core set of aesthetic antonyms from the collected ones, the 20 participants were asked to select some pairs of aesthetic antonyms from Table 2 that can describe their general feelings at first glance. The most frequently mentioned 8 pairs of semantic antonyms were selected as the core set for model building as shown in Table 3.

### 3.2. Clustering of Aesthetic Antonyms

The aim of this experiment was to cluster the 8 pairs of aesthetic antonyms into three groups. Each cluster corresponds to a layer of the proposed hierarchical model. First, 100 questionnaires were prepared that include 3 questions as mentioned below and the aesthetic antonyms as listed in Table 3. Second, 100 participants were recruited (45 males and 55 females, age range 16–24) to categorize the 8 pairs of aesthetic antonyms into three clusters according to the following three questions. Finally, the 8 pairs of aesthetic antonyms were grouped into 3 clusters by participants.

 
*Question 1*: which aesthetic antonyms can be used to describe the initial perceptual feeling of the texture at first glance?
 
*Question 2*: which aesthetic antonyms can reveal the nature of the thing that makes the visual texture itself?
 
*Question 3*: which aesthetic antonyms can describe your feeling when you are asked to make a decision about the visual texture?

The clustering result is shown in Table 4. The cumulative percentage, such as 91%, refers to the proportion of the participants who cluster the aesthetic antonym to the given question.

### 3.3. Aesthetic Evaluation of Visual Textures

**Figure 2:**
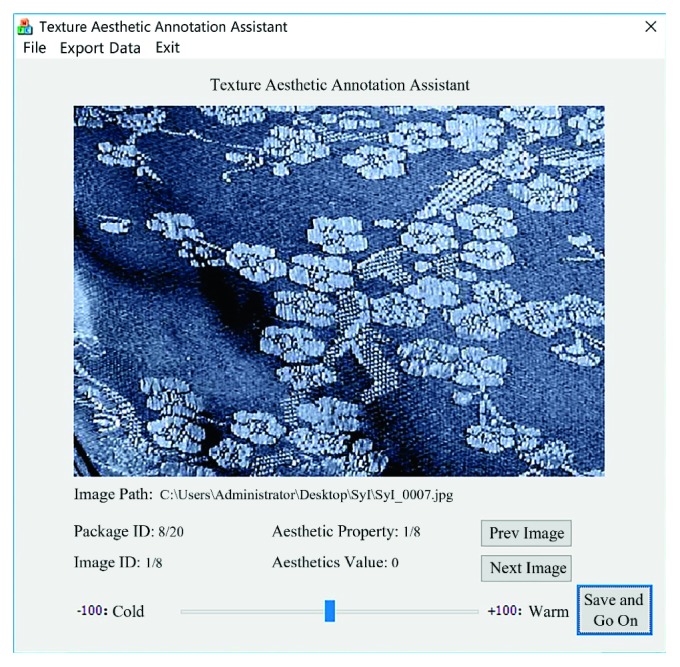
Texture aesthetic annotation assistant.

Twenty undergraduate students of Jiangnan University (10 males and 10 females, age from 19 to 23) served as participants to rate 151 visual textures against the eight pairs of aesthetic antonyms as listed in Table 3. A special tool called Texture Aesthetic Annotation Assistant was developed to help to complete the evaluation procedure, which is shown in Figure 2. When a visual texture is selected, it will be displayed under a gray background. By dragging a scroll bar at the bottom, the participants can evaluate each visual texture in a continuous rating scale within the interval (−100, 100), which is useful for a continuous regression model. When the semantic differential experiment was completed, the ratings for the same visual texture evaluated by the 20 participants were averaged after removing outliers and used as the final rating to construct a prediction model for aesthetic emotions.

## 4. Definition of the Model Structure

**Figure 3:**
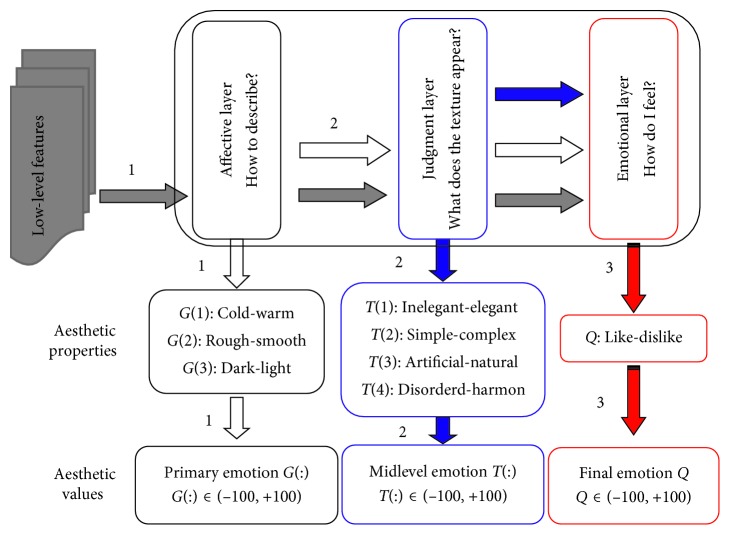
Model structure of aesthetic perception of visual texture. The model consists of three layers, which correspond to the three questions mentioned in Section 3.2.

To some extent, the model building of aesthetic perception of visual textures is a special case of machine learning, in which a model can be built to bridge the gap between the low-level textural features and the high-level aesthetic emotions. So, the general methods, such as neural network, probability estimation, genetic algorithm, support vector machine, subset regression, ridge regression, bagging prediction, boosting prediction, and random forest, can also be used to build a model to connect low-level texture features to high-level aesthetic emotions [24, 25]. However, the models based on machine learning without a better understanding of psychological and neurocognitive events are unsuitable to interpret the relationship between the low-level features and the high-level aesthetic properties. Based on the achievements of the neural foundations of neuroaesthetics, particularly the brain's specialized systems for aesthetic judgment, we generated a model with a hierarchical structure [9, 26–29]. The structure of the hierarchical feed-forward model of aesthetic texture perception is shown in Figure 3. Three functional layers constitute the hierarchical model for visual aesthetic perception, which corresponds to the three questions proposed in Section 3.2.

As described in Section 2, the low-level feature set, *M*_*p*_={*A*_*i*_^*p*^, *B*_*j*_^*p*^, *C*_*k*_^*p*^,…} of the *p*^th^ visual texture will be calculated, where *i*=1,2,…, *n*, *j*=1,2,…, *s*, *k*=1,2,…, *t* represents the number of the different texture feature subsets *A*, *B*, and *C*, etc. After feature selection, each visual texture is represented by 8 low-level features. The aesthetic values of the affective, judgment, and emotional layers of the *p*^th^ visual texture are represented by *G*_*p*_={*G*(1)^*p*^; *G*(2)^*p*^; *G*(3)^*p*^}, *T*_*p*_={*T*(1)^*p*^; *T*(2)^*p*^; *T*(3)^*P*^; *T*(4)^*p*^}, and *Q*_*p*_={*Q*^*p*^}, respectively. Based on the ideas conveyed in Figure 3, we employ 6 activation functions to construct the three perception channels.

Perception model of the affective layer:

(1) $$\begin{array}{c} G=F_{1}\left(M\right)+R_{0}. \end{array}$$

Perception model of the judgment layer:

(2) $$\begin{array}{c} T=F_{2}\left(M\right)+F_{3}\left(G\right)+R_{1}. \end{array}$$

Perception model of the emotional layer:

(3) $$\begin{array}{c} Q=F_{4}\left(M\right)+F_{5}\left(G\right)+F_{6}\left(T\right)+R_{2}, \end{array}$$

where *F*_1_, *F*_2_, *F*_3_, *F*_4_, *F*_5_, and *F*_6_ are the 6 activation functions that are linear or nonlinear; *R*_0_, *R*_1_, and *R*_2_ refer to the emotion thresholds, which are the minimal value of the ratings of the aesthetic properties; and the symbol “+” indicates emotions accumulated through different perception stages as demonstrated in Figure 3.

## 5. Model Building for Aesthetic Perception

Before model building, data smoothing and normalization were performed on the selected feature set to ensure model robustness. Then, the feature matrix of the 151 visual textures was divided into two sets. The training set included 90% of the total number of textures and was used for model building. The test set was used to evaluate the performance of the models built on the training set to measure the expected quality of new textures. When the target expressions are defined by Equations (1)–(3), the basic, trigonometric, and exponential functions are selected in the formula building blocks of the Eureqa Desktop [30]. In detail, the basic functions include addition, subtraction, multiplication, division, and the constant operation. The trigonometric functions include sine, cosine, and tangent functions. The exponential functions include exponential, natural logarithmic, factorial, power, and square-root functions.

Three parameters, referred to as complexity, mean absolute error, and correlation coefficient were used to evaluate the constructed model. The model with the greatest correlation coefficient and the least mean absolute error was considered the best. The model complexity is defined as the VC-dimension, proposed by Vapnik Cherkassky in the statistical learning theory [31]. Thus, the models selected for the eight pairs of aesthetic properties distributed in the hierarchical feed-forward model are as follows. The corresponding evaluation parameters are demonstrated in Table 5.

(4) $$\begin{array}{c} G\left(1\right)=-8094.8087+812.5336\cdot f_{8}+45616.6421\cdot f_{9}+912.3156\cdot f_{10}-\frac{12.2778}{f_{1}}-193.0201\cdot f_{1}\cdot f_{9}-3851\cdot f_{8}\cdot f_{9}-5152.5354\cdot f_{9}\cdot f_{10}, \end{array}$$

(5) $$\begin{array}{c} G\left(2\right)=-2.4097+2.4427\times 10^{-3}\cdot f_{7}, \end{array}$$

(6) $$\begin{array}{c} G\left(3\right)=-17383.3439+1632.0393\cdot f_{1}-8.4352\cdot f_{3}+4091.5619\cdot f_{10}-179.9276\cdot f_{1}\cdot f_{7}-1740.2929\cdot f_{1}\cdot f_{9}-239.9238f_{10}^{2}, \end{array}$$

(7) $$\begin{array}{c} T\left(1\right)=-5802.8782-2.6621\cdot f_{1}+1376.0294\cdot f_{10}+G\left(3\right)+0.5044\cdot f_{3}\cdot G\left(3\right)-f_{8}\cdot G\left(3\right)f_{1}\cdot f_{3}\cdot G\left(1\right)-81.5644\cdot f_{10}^{2}, \end{array}$$

(8) $$\begin{array}{c} T\left(2\right)=-120.8679+18.7771\cdot f_{10}+6.2114\cdot f_{3}\cdot f_{4}-16.5658\cdot f_{2}\cdot f_{4}-\frac{5.0154}{f_{4}\cdot f_{9}}+14.4728\cdot G\left(1\right)\cdot f_{9}^{2}, \end{array}$$

(9) $$\begin{array}{c} T\left(3\right)=-525.3141+22064.1366\cdot f_{4}+f_{7}+61.6075\cdot f_{10}-92.5762\cdot f_{1}\cdot f_{8}-152.7179\cdot f_{1}\cdot f_{2}+228.3617\cdot f_{9}^{2}-5175.2472\cdot f_{3}\cdot f_{4}\cdot f_{10}+303.4996\cdot f_{3}\cdot f_{4}\cdot f_{10}^{2}, \end{array}$$

(10) $$\begin{array}{c} T\left(4\right)=4.3881\times 10^{3}+1.8217\cdot G\left(2\right)+71.5439\cdot f_{9}+0.8139\cdot f_{7}, \end{array}$$

(11) $$\begin{array}{c} Q=38.4474-10.7260\cdot f_{1}-57.6083\cdot f_{8}+0.0914\cdot T\left(3\right)+1.4546\cdot f_{1}\cdot G\left(1\right), \end{array}$$

where *f*_*i*_, *i*=1,2,…, 10 represents the 10 features selected.

We determined that the majority of the built models are nonlinear models, except for Equation 5. We use more nonlinear terms for model building when compared with a previous study [17, 18], although linear models are sufficient to bridge the gap between level texture features and high-level aesthetic properties. When nonlinear models are selected, the prediction error will obviously decrease; however, the model complexity will sharply increase when compared with the linear model. In fact, 13 different nonlinear terms were selected for model building in Eureqa, which automatically selects the terms most feasible for establishing a high-quality model through cross-validation. According to Equations (1)–(3), the dimensionalities of the feature vectors are 10, 13, and 17, respectively. To some extent, the constructed models are much simpler beyond our expectations. According to Equations (4)–(11), only some of the 10 selected features are involved, although we use four algorithms to extract 106 features for each visual textures.

Additionally, Equations (4)–(11) indicate that the high-level aesthetic properties in the affective, judgment, and emotional layers all cover low-level texture features. According to Equations (7), (8), (10), and (11), the high-level aesthetic properties in judgment and emotional layers cover the aesthetic properties in the lower level layer. Interestingly, *G*(1) and *G*(3) are important variables of the models for *T*(1), *T*(2), *T*(3), *T*(4), and *Q*. The low-level texture features, such as the mean of saturation in color space, contrast of GLCMs, and *L*^1^ norm of wavelet coefficients extracted from vertical subband at level 1, have direct influence on high-level aesthetic feelings.

## 6. Conclusions

We propose a white-box model with high interpretability to bridge the gap between low-level statistical features and high-level aesthetic emotions. Both the texture analysis algorithms and model building method introduced in this work are generalized to all visual textures.

First, we used four different algorithms to calculate the low-level texture features, including color features, statistical moments of gray-level co-occurrence matrix, Tamura texture features, and wavelet energy signatures in the frequency domain, to fully represent the characteristics of visual textures. To simplify the model complexity and improve the learning speed and generalization capacity of the induced model, we used neighborhood component analysis for robust feature selection before model building.

Second, during the psychologic semantic differential experiment, more than 100 participants were recruited to complete the selection of suitable aesthetic antonyms and aesthetic evaluation of the visual textures when the core set of aesthetic properties are decided.

Finally, a white-box model with hierarchical feed-forward structure was proposed, which has obvious interpretation of internal structure to explain interrelations between low-level textural features and high-level aesthetic emotions. Then, we used nonlinear functions to complete model building. Experiment results indicate that the proposed model has high robustness and prediction accuracy. We also found that some computational low-level features correlate well with the high-level aesthetic emotions. To some extent, this research suggests that the aesthetic perception of visual textures is sufficiently universal to be predictable when a cognitive model is built by combining bioinformatics and neuropsychological and neurobiological science.

The major limitation of this research is the number of texture samples that have been evaluated. Additionally, the participants are limited to a very narrow range of ages and educational background. Future research will focus on the influence of the number of texture samples and the educational background of participants recruited in the semantic differential experiment.

## Figures and Tables

**Table 1:** Selected features through neighborhood component analysis.

Index	Weight	Category	Parameters	Name
*f*_1_	4.8884	Color characteristics	Mean of saturation	Mean of saturation
*f*_2_	4.2978	GLCMs	*d *=* *8, *θ *=* *45°	Contrast
*f*_*3*_	3.3620	GLCMs	*d *=* *6, *θ *=* *45°	Contrast
*f*_4_	3.0538	GLCMs	*d *=* *8, *θ *=* *135°	Contrast
*f*_5_	2.9077	GLCMs	*d *=* *8, *θ *=* *90°	Homogeneity
*f*_6_	2.4637	GLCMs	*d *=* *4, *θ *=* *45°	Contrast
*f*_7_	1.8981	Tamura texture	—	Coarseness
*f*_8_	1.8723	Tamura texture	—	Directionality
*f*_9_	1.7262	Wavelet-based energy	Horizontal subband at level 1	*L* ^2^ norm
*f*_10_	1.5843	Wavelet-based energy	Vertical subband at level 1	*L* ^1^ norm

**Table 2:** The collected 20 pairs of aesthetic antonyms.

Aesthetic antonym	Aesthetic antonym	Aesthetic antonym	Aesthetic antonym	Aesthetic antonym
Warm-cold	Hard-soft	Strong-weak	Dynamic-static	Natural-artificial
Rough-smooth	Fresh-muddy	Dismal-cheerful	Sophisticated-coarse	Comfortable-uncomfortable
Dark-light	Mussy-harmonious	Modern-ancient	Random-regular	Like-dislike
Wet-dry	Gallant-plain	Simple-complex	Inelegant-elegant	Love-hate

**Table 3:** The core set of aesthetic antonyms.

Aesthetic antonym	Aesthetic antonyms
Warm-cold	Inelegant-elegant
Rough-smooth	Simple-complex
Dark-light	Artificial-natural
Mussy-harmonious	Like-dislike

**Table 4:** The clustering of aesthetic antonyms (%).

Index	Aesthetic antonym	Question 1	Question 2	Question 3
1	Cold-warm	91	7	2
2	Rough-smooth	88	11	1
3	Dark-light	86	12	2
4	Mussy-harmonious	13	86	1
5	Inelegant-elegant	2	90	8
6	Simple-complex	8	90	2
7	Artificial-natural	11	76	13
8	Like-dislike	1	8	91

**Table 5:** Evaluation parameters of the built models.

Aesthetic property	Mean absolute error	Correlation coefficient	Complexity
G(1)	0.7046	0.9951	36
G(2)	0.0001	0.9215	5
G(3)	1.2581	0.9827	35
T(1)	0.6258	0.9952	31
T(2)	0.7692	09954	32
T(3)	1.5154	0.9707	35
T(4)	0.7835	0.9876	19
Q	10.6753	0.7977	5

## Data Availability

The data used to support the findings of this study are available from the corresponding author upon request.
